# Low-Cost Investment with High Quality Performance. Bleaching Earths for Phosphorus Reduction in the Low-Temperature Bleaching Process of Rapeseed Oil

**DOI:** 10.3390/foods9050603

**Published:** 2020-05-08

**Authors:** Barbara Łaska-Zieja, Damian Marcinkowski, Wojciech Golimowski, Gniewko Niedbała, Ewelina Wojciechowska

**Affiliations:** 1 Institute of Technology and Life Sciences, Hrabska 3, 05-090 Raszyn, Poland; b.laska@itp.edu.pl (B.Ł.-Z.); damian.marcinkowski@outlook.com (D.M.); 2 Wroclaw University of Economics and Business, Komandorska 118-120, 53-345 Wroclaw, Poland; wojciech.golimowski@ue.wroc.pl; 3 Institute of Biosystems Engineering, Faculty of Agronomy and Bioengineering, Poznań University of Life Sciences, Wojska Polskiego 50, 60-627 Poznań, Poland; 4 Norwegian Institute of Bioeconomy Research (NIBIO), Postboks 115, 1431 Ås, Norway; ewelina.wojciechowska@nibio.no

**Keywords:** rapeseed oil, bleaching process, bleaching earth, phosphorus, food technology

## Abstract

Rapeseed oils are a valuable component of the diet. Mostly, there are refined oils deprived of valuable nutrients in the market, hence in recent times cold-pressed and unrefined oils have been available and popular among consumers. However, the low yield of this oil makes this product expensive. The aim of the study was to analyse the effectiveness of phosphorus reduction in crude oils, cold- and hot-pressed in the low-temperature bleaching process. Eight market-available bleaching earths was compared. The effectiveness of 90% was found with 2% (m/m) of Kerolite with hydrated magnesium silicate. An increase in the share of earths to 4% (m/m) resulted in the effectiveness of phosphorus reduction >90% in seven out of eight analysed cases. Bentonite activated with acid with the lowest MgO content was characterised by low efficiency <64%. The research shows that the effectiveness of phosphorus reduction was significantly affected by the composition of earths applied in the bleaching process at ambient temperature. The results of research confirm the high effectiveness of the process as it is not necessary to heat up the oil before the bleaching process. This method is recommended for existing and new industrial plant for two-stage rapeseed oil pressing.

## 1. Introduction

Rapeseed oil is widely produced and consumed in Europe and is the third most important vegetable oil after palm and soybean oils in the world [1,2]. In the market there are available pressed, unrefined oils containing valuable nutrients. Rapeseed oil has a lower concentration of saturated fatty acids (5−10%), higher content of monounsaturated fatty acids (44−75%), and moderate content of α-linolenic acid (9−13%) [3]. The production of cold-pressed oil is inefficient, while the production of hot-pressed oil results in its dark colour and the presence of phospholipids that are harmful to health. The solution is oil refining, as a result both phospholipids and valuable nutrients are removed.

On an industrial scale, phosphorus is removed from the oil in the degumming process. The application of aqueous acid solutions results in removing phospholipids. The process requires high-tech facilities. In case of the low-temperature bleaching process, no additional facilities are needed, but only minor modifications are required. The process does not require the use of advanced equipment for precise separation of the fractions. The results of research can be used directly in the oil production.

The amount of phospholipids in the oil primarily depends on the way it was obtained. Cold-pressed rapeseed oil can contain from 14.57 ppm to as much as 186.4 ppm of phosphorus, whereas hexane-extracted oil can have 482.1 ppm [4,5]. Crude soybean oil can contain up to 200 ppm of phosphorus, coconut oil - up to 400−500 ppm [6]. The amount of phospholipids in the oil depends on the pressing temperature [7]. Research by Rotkiewicz and Konopka [8] upon rapeseed conditioning showed that the cold-pressed rapeseed oil had 46 ppm of phosphorus and oil from seeds warmed up to 60 °C contained 83 ppm. However, with a temperature increasing from 80 to 100 °C, the phosphorus content in the oil increased from 125 ppm to over 300 ppm [9]. A decrease in moisture of seeds also lead to an increase in the phosphorus content in the vegetable oil [10,11]. The phosphorus content in the sunflower oil pressed from seeds with a moisture of 6% equaled to 85 ppm, for seeds with a moisture of 5%–106 ppm and for seeds with a moisture of 2%–23 ppm [12].

The purification of crude vegetable oil from undesirable substances adversely affects the quality and durability of fat (phospholipids, free fatty acids, sterols, waxes, oxidation products, water, aromatic compounds, pigments) and is carried out in a refining process that involves degumming, deacidification, bleaching and deodorization [13,14,15,16]. Crude vegetable oil comprises phospholipids in two forms: hydratable and non-hydratable [14,17]. In the refining process, phospholipids are removed at the degumming stage [18,19]. For example, solvent-extracted oil contains 529 ppm of phosphorus, and after degumming this amount was reduced to 12.2 ppm [20]. The degumming process involves adding about 2% of water at a temperature of about 80 °C to the oil and intensive stirring. This process allows removing hydratable phospholipids from the oil in the form of gums (about 0.03−0.3%) containing lecithin. This gum is then easily separated from the oil by centrifugation [14,21,22]. On the other hand, non-hydratable phospholipids can be removed from the oil with the use of small amounts of organic acids, including citric, malic or phosphoric ones [23,24,25]. In the oil, they produce insoluble metals, salts and hydrated phospholipids which can be easily removed [14,22]. In the two-stage water and phosphoric acid degumming process, the amount of phospholipids in rapeseed and sunflower oils was reduced to 10 ppm [25]. The process that finally removes phospholipids from the oil is bleaching. It also removes coloured compounds such as chlorophyll and carotene, soap, sulphur, metals and oxidation products from the oil [14,26]. It improves the organoleptic properties of vegetable oil and its durability. Bleaching is the adsorption of undesirable compounds by means of bleaching earths or activated carbon [14]. Bleaching earths from mineral clays consisting of aluminium silicates such as calcium montmorillonite (bentonite) [27], attapulgite, sepiolite [28,29] are most commonly applied in the process of bleaching vegetable oils. Vegetable oils are primarily cleaned with bleaching earths produced from bentonite due to their good discolouring and deacidifying properties and their effectiveness in removing other impurities from the oil. Bleaching earths are physically and chemically activated in order to increase their sorption surface [14,30]. The effectiveness of montmorillonite-based bleaching earths (with 2% (m/m) in the oil) in the purification of filtered rapeseed oil from phosphorus was determined to be over 60% [31]. After refining, the rapeseed oil contained between 2.5–22 ppm of phosphorus [32].

The color of vegetable oil is essentially due to α- and β-carotenes, which are mostly removed by an adsorption process called bleaching [33,34]. This process also aims to remove other pigments (e.g., Chlorophylls), metals (iron and copper), soaps and oxidation products [33]. The bleaching process is primarily intended to reduce the amount of carotene and chlorophyll, which are responsible for the color of the oil. It was observed that the side effect is the reduction of phosphorus correlated with the oil color; however, the soil porosity did not affect the efficiency of adsorption [34]. Adsorbents such as activated carbon, silica and acid activated clays are widely used in industry [35].

Phospholipids decrease oil stability against oxidation reactions, influence the organoleptic properties, and they are harmful in technological processes especially oil hydrogenation [36]. It is important to recognize significant amount of phosphate content delivered in food/beverages [37,38] and attempting to obtain food products containing the lowest possible phosphate doses. Our research focused on the preparation of rapeseed oils containing the lowest possible phosphorus content using bleaching earths.

The research was conducted to verify phenomenon of the adsorption of impurities and colour compounds from vegetable oils by bleaching earths. The aim of experiment was to determine the effect of the composition of bleaching earths in the low-temperature bleaching process on the oil quality. The phosphorus content was the parameter classifying the oil quality. The two-stage reduction of phosphorus in hot- and cold-pressed rapeseed oil was determined.

## 2. Materials and Methods

**Figure 1:**
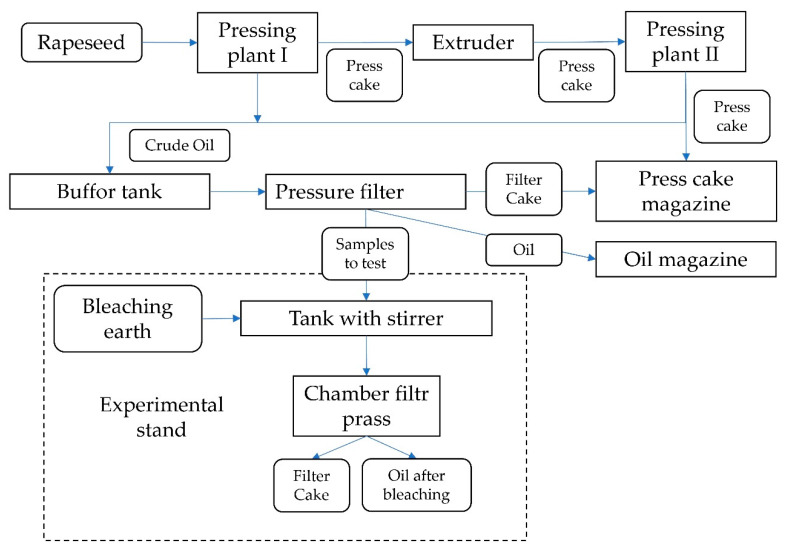
Diagram of oil production technology and experimental stand.

Rapeseed oil was obtained by two-stage pressing method (cold and hot) in an industrial plant, in Poland in Koźmin Wlkp. with an annual production capacity of 30 thousand tonnes of rapeseed. At the first stage, industrial seeds (impossible to identify variety) were pressed without preliminary heating and then the press cake was extruded at 120 °C and then re-pressed. Oils from both pressing plants were mixed in a buffer tank, then filtered on a pressure filter. Samples from each batch of the filtered oil (10 kg each, in 3 replicates) were collected. For each sample, 2%, 4%, 6% (m/m) of bleaching earth from eight different producers were added, respectively. The oil with a temperature of 28 °C ± 3 °C was mixed with the earth for 30 min and then it was filtered with the use of a chamber filter press with a filter area of 0.24 m^2^ [39]. After filtration, filter cake was weighed and the loss of oil during filtration was determined. For the oil samples collected before and after the refining process, the analysis of phosphorus content was carried out by an accredited laboratory in accordance with the PB-69/IPC standard, version 2, dated 18 September 2012, method of inductively coupled plasma optical emission spectroscopy (ICP-OES). Figure 1 describes the diagram of the experimental stand.

Various bleaching earths from Europe and the USA were applied in the oil refining process. The parameters of bleaching earths were shown in Table 1.

The effectiveness of phosphorus reduction was calculated from the following Formula (1):

(1) $$S_{r}=\frac{F_{p}-F_{b}}{F_{p}}\times 100\;\left[\%\right]$$

where:

S_r_ = the effectiveness of phosphorus reduction of a specific bleaching earth used to bleach rapeseed oil [%],
F_p_ = the amount of phosphorus in rapeseed oil before purification by bleaching [mg/kg],
F_b_ = the amount of phosphorus in rapeseed oil purified by bleaching [mg/kg].

The amount of oil loss was calculated from the following Formula (2):

(2) $$S_{t}=\frac{m_{b}-m_{z}}{m_{o}}\times 100\;\left[\%\right]$$

where:

S_t_ = the amount of oil in the filter cake with respect to the mass of filtered oil [%],
m_b_ = the mass of filter cake [kg],
m_z_ = the mass of bleaching earth used for filtration [kg],
m_o_ = the mass of filtered oil [kg].

The results of research were subject to the statistical analysis of the correlation matrix and t tests for dependent samples. The share of oxides, bulk density and pH of bleaching earths were considered as independent variables, whereas the level of phosphorus reduction and the amount of oil loss were assumed to be dependent variables. 

## 3. Results and Discussion

The results of effectiveness of phosphorus reduction from equation No 1 in the low-temperature bleaching process were shown in Table 2.

The percentage share and type of bleaching earth significantly affected the reduction of total phosphorus. The analysis of the results proved that the bleaching earth P2 had the lowest effectiveness of phosphorus reduction. In other cases, as little as 4% of bleaching earth in relation to the oil mass was characterized by effectiveness above 90%, obtaining the oil with a phosphorus content below 30 ppm. The highest effectiveness of phosphorus reduction was observed with 6% m/m of the bleaching earth, where the phosphorus content after the bleaching process was <1 ppm. The use of earth P6 is economically justified because of the high effectiveness of 90.9% with the share of 2% m/m. The phosphorus content in the oil was lowered to 10 ppm.

The rapeseed oil market classifies oil into unrefined oil up to 30 ppm and refined oil up to 300 ppm of phosphorus. The oil up to 30 ppm of phosphorus can be obtained in the low-temperature oil pressing plant or after the oil degumming process at temperatures above 80 °C [32]. The threshold of 30 ppm of phosphorus was adopted for the optimization analysis of the minimum application of bleaching earths. The normal distribution of phosphorus in the tested oils was described by a polynomial function and the minimum amount of bleaching earths application was calculated and the amount of oil loss in this process was determined. The results of research shown in Table 3 can be used for economic analysis of the low-temperature bleaching process in the oil production technology.

Based on the results shown in Table 3, significant differences in the amount of applied bleaching earths and the amount of loss resulting from the oil absorption were proven. The most favourable for use were earths P6 and P7 which had the highest effectiveness and the lowest oil absorption. For example, by using the sample P6, 17.9 kg of the bleaching earth could be used to filter 1 Mg of the oil and the loss would be 4.5 kg of the oil. At current prices, the bleaching cost equals to 2% of the oil price.

The bleaching earths applied in the research were characterized by different elementary composition and properties. The impact of particular components on the effectiveness of phosphorus reduction were statistically analyzed (Table 4).

The significant phosphorus reduction can be observed in all cases. The impact of pH was correlated positively, especially with a higher share of bleaching earths. Positive correlation between PH and 3-monohropropenem-1,2-dio esters and glycidyl esters, contents in palm oil and phosphorus was significant [36]. The soil density had a significant impact. Silicon oxide, aluminium oxide and sodium oxide had an adverse effect on the phosphorus reduction. Iron oxide, magnesium oxide and calcium oxide had a positive effect. The earth samples (Table 1) P6 and P7 were characterised by the highest share of magnesium oxides 30.5% and 20%, respectively. Moreover, the sample P7 had the highest share of iron oxides (15%) in comparison with other bleaching earths.

The use of bleaching earths in oil refining was the subject of many studies and were used in the bleaching process of vegetable oils to remove impurities, i.e., oxidants, phosphorus, trace amounts of heavy metals and dyes. Published studies confirm the thesis that the type of used bleaching earths had a significant impact on reducing the level of impurities in vegetable oil [40,41]. Depending on the activation method, the bleaching earths has different properties, and thus differ in the bleaching efficiency [42].

Refining of edible oils usually involves four stages: degumming, neutralization, bleaching and deodorization [42]. Bleaching takes place with the help of porous materials characterized by high adsorption, which are called bleaching earths [43]. Most of the commonly used bleaching earths can be classified as bentonites [44] and kaolinites [45].

The technique of mechanical removal of impurities from oil with bleaching earths can be compared to membrane techniques. Research of Sehn, Gonçalves, Ming [46] have shown a high efficiency (95.5%) of phosphorus removal from oil using polymer membranes. The use of phospholipidase C (PLC) is also effective, resulting in a crude oil with phosphorus content 7.34 ± 0.39 mg/kg [47].

Ramanaiah [48] described the possibility of using spent bleaching earths (RSBE) as an adsorbent to remove fluoride ions from aqueous solutions and ultimately from drinking water. Spent bleaching earths were regenerated by using acids and bases. The research was carried out in a batch system, where the effect of added bleaching earth, pH level and contact time with the adsorbent on the concentration of fluoride ions in the tested solutions was examined. The maximum level of absorption was obtained at pH = 7 and when the contact time with the adsorbent was 180 min. The maximum level of fluoride ion adsorption, calculated per 1 g of regenerated bleaching earth, was 0.6 mg/g. Analysis of the kinetics of the adsorption process allowed to determine that this process occured at a speed of 0.64 mg/g·min^0.5^. 

Plata described the usage of regenerated bleaching earth in the filtration process of biodiesel obtained from vegetable oil. Bleaching earth was reactivated by washing with hexane and by treatment with 0.1 M HCl at 500 °C [49].

## 4. Conclusions

The high effectiveness of bleaching earths can be determined when removing phosphorus from rapeseed oil in the low-temperature bleaching process. The presence of metal oxide in bleaching earths was significant. Bleaching earths enriched with iron and magnesium oxides had the best absorption properties of phospholipids. In case of the two-stage pressed oil at temperatures initially 50 °C, then 120 °C, rapeseed oil with parameters of 30 ppm of phosphorus can be achieved by using less than 2% (m/m) of bleaching earth with respect to the oil. The low-temperature bleaching solution can be recommended for small oil plants that are unable to invest in a professional rapeseed oil refining plant.

## Figures and Tables

**Table 1:** Selected physicochemical properties of bleaching earths used in the process of oil refining.

Parameter	Bleaching Earths							
	P 1	P 2	P 3	P 4	P 5	P 6	P 7	P 8
**Moisture (105°C), % (m/m)**	11−19	10−12	14.0−17.5	14.0−17.5	about 15	4−6	12	14
**Mineral**	Acid-activated bentonite		Sulphuric acid-activated bentonite	Bentonite	Active carbon bentonite	Kerolite - hydrated magnesium silicate	Attapulgite	
pH value	5.0	3.5	3.5	7.0	5.0	6.0	8.0	8.5
Bulk density, g/dm^3^	400	500	650	650	800	550	350	450
Particle size > 75 µm, %	about 22	about 94	-	-	-	6	-	-
Particle size >45 µm, %	about 45	about 70	74.0−80.0	80.0−86.0	70	24	35	22
**Composition (% share):**								
**SiO_2_**	75.6	69.6	68.5	67.5	67.0	53.5	60	65
**Al_2_O_3_**	11.4	11.8	11.0	12.5	12.0	4.0	3	6
**Fe_2_O_3_**	4.4	3.4	3.5	3.0	4.5	1.5	15	13
**MgO**	3.2	2.6	6.0	6.5	6.5	30.5	20	15
**CaO**	3.1	0.7	2.5	3.0	2.0	0.7	0.5	0.5
**Na_2_O**	0.4	0.4	0.2	0.3	0.1	0.3	0.05	0.05

**Table 2:** Results of effectiveness of reduction phosphorus and oil loss in the bleaching process.

Bleaching Earth	Crude Oil		2% (m/m)		4% (m/m)		6% (m/m)	
	Phosphorus Share [mg/kg]	Oil Mass [kg]	S_r_ [%]	S_t_ [%]	S_r_ [%]	S_t_ [%]	S_r_ [%]	S_t_ [%]
P1	97.4 ± 9.7	10 ± 0.02	56.8	1.6	91.3	2.6	98.2	4.2
P2	95.4 ± 9.5	10 ± 0.02	40.8	1.3	59.0	1.5	63.3	2.0
P3	145.0 ± 15	10 ± 0.02	81.6	0.6	93.1	1.6	97.0	3.3
P4	145.0 ± 15	10 ± 0.02	87.4	1.4	94.9	2.5	97.1	3.7
P5	150.0 ± 15	10 ± 0.02	44.2	1.3	90.7	2.7	98.1	4.6
P6	110.0 ± 16	10 ± 0.02	90.9	0.5	95.4	1.1	95.9	2.5
P7	110.0 ± 16	10 ± 0.02	77.1	0.6	98.2	1.9	99.1	2.5
P8	109.0 ± 11	10 ± 0.02	49.2	1.1	91.2	1.4	95.9	2.0

**Table 3:** Optimal amount of applied bleaching earths and the amount of loss in the low-temperature bleaching process.

Bleaching Earth	The Polynomial Equation Describing the Normal Distribution of Measurement Points *	R^2^	*x → Min Where y = 30 ppm of Phosphorus	Oil Loss During the Bleaching Process [%] (m/m)
P1	y = 3.04x^2^ − 34.27x + 97.67	0.99	2.56	2.05
P2	y = 2.18x^2^ − 22.98x + 94.99	0.99	-	-
P3	y = 7.15x^2^ − 65.10x + 142.41	0.97	2.32	0.74
P4	y = 7.83x^2^ − 68.93x + 141.51	0.96	2.14	2.05
P5	y = 3.46x^2^ − 46.30x + 153.12	0.99	3.66	2.38
P6	y = 6.21x^2^ − 53.36x + 105.47	0.95	1.79	0.45
P7	y = 5.24x^2^ − 48.93x + 108.03	0.99	2.05	0.62
P8	y = 3.03x^2^ − 36.16x + 110.65	0.99	2.98	1.64

*x – mass of bleaching earth with respect to % of the oil mass (m/m); y – share of phosphorus in the oil in ppm

**Table 4:** Statistical analysis and the correlation of components of bleaching earths in phosphorus reduction in the oil.

Share of Bleaching Earth	pH	Density	SiO_2_	Al_2_O_3_	Fe_2_O_3_	MgO	CaO	Na_2_O
2%	0.21	0.02	−0.52	−0.34	0.17	0.52	0.17	−0.04
4%	0.54	0.04	−0.35	−0.40	0.26	0.25	0.25	−0.49
6%	0.48	0.10	−0.20	−0.28	0.24	0.34	0.34	−0.49
